# Minnesota State Veterinary Medical Association

**Published:** 1901-02

**Authors:** K. J. McKenzie

**Affiliations:** Secretary and Treasurer


### MINNESOTA STATE VETERINARY MEDICAL ASSOCIATION.

THE eighth semi-annual meeting of the Association was called to order by President S. H. Ward at 1.30 P.M., at the Metropolitan Hotel, St. Paul, Minn., January 10, 1901.

The following members responded to the roll-call: Drs. C. C. Lyford, M. H. Reynolds, S. D. Brimhall, R. Price, L. Hay, K. J. McKenzie, S. H. Ward, J. G. Annand, J. A. Hanisch, J. N. Gould, J. W. Gould, J. J. McLaughlin, B. A. Pomeroy, H. C. Lyons, J. S. Butler, H. C. Peters, M. J. Sexton, F. H. Farmer, J. P. Foster, R. La Pointe, and D. M. McDonald.

The minutes of the last meeting were read and approved. President Ward then gave his address, going over the work done by the Association pretty thoroughly and dealing with the work it should take up in the future. Among other things the doctor spoke of a bill to be introduced at the present session of the Legislature to reimburse owners of condemned glandered horses, and asked the support of the members in its behalf. The doctor thought that such horses should be appraised at about two-thirds their value, and in order for the owner to secure the indemnity he must give satisfactory proof that the condemned animal has been owned in this State for at least three months prior to being condemned. Dr. Ward also spoke of our State Examining Board, and thought it should be composed of qualified veterinarians who were members of this Association.

The retiring President, Dr. Ward, then thanked the Secretary and members for their hearty support during his term of office.

The Treasurer's report was then read and accepted. The following gentlemen were then duly elected to membership: Drs. J. M. Lambert, of St. Peter; Oscar Rydell, of Wheaton; F. A. Ilstrup, of Wilmer; and H. A. Hela, of Cokato.

Dr. S. D. Brimhall next reported for the Committee on Infectious Diseases. Eight different outbreaks of hemorrhagic septicæmia had been reported since the last meeting; anthrax, only one outbreak, in which two cases were reported; cerebro-spinal meningitis had been reported in two instances; several cases of rabies had been reported; two outbreaks of foot rot in cattle had been investigated and two others reported.

Dr. Reynolds gave his report on recent advances in veterinary surgery, which proved quite lengthy and dealt extensively with matters pertaining to recent advances. Dr. Lyford reported for the Committee on Legislation; quite a lively discussion followed. Dr. Price thought we should call the attention of the Horseshoers' Union to the fact that there is a law in this State governing the practice of veterinary medicine and surgery.

On motion the several reports were accepted and committees discharged. Dr. Price then read his paper on "Rabies," which proved very interesting, as a great deal of it was taken from actual experience with cases in the doctor's practice, and numerous photographs of different cases were passed among the members. Dr. Lyford led the discussion of Dr. Price's paper and gave his experience in autopsies while in London with Dr. Fleming.

The following officers were elected for the ensuing year: President, Dr. J. N. Gould, Worthington; First Vice-President, Dr. R. Price, St. Paul; Second Vice-President, Dr. J. J. McLaughlin, Blue Earth City; Secretary and Treasurer, Dr. K. J. McKenzie, Northfield; Trustees, Drs. L. Hay, J. W. Gould, and H. C. Peters.

On motion it was decided that Northfield should be the place for holding the next meeting. The meeting then adjourned for supper.

At 7.30 P.M. J. P. Foster read his paper on "The Management of Brood Mares." A lengthy discussion followed, touching on the best purgatives to be given a mare in foal and the treatment of mares about to abort.

Dr. H. C. Peters next read his paper entitled "A Non-classified Disease in Horses." Dr. Annand reported similar cases occurring in Beltrami County, and Dr. Ward reported cases of a similar nature met with while practising in Manitoba. Dr. Gould, the newly elected President, then appointed the following committees: Colleges—Dr. D. M. McDonald. Infectious Diseases—Dr. S. D. Brimhall. Finance—Dr. J. J. McLaughlin. Legislation—Drs. M. H. Reynolds, H. C. Lyons, and M. J. Sexton. Recent Veterinary Literature—Surgery, Dr. J. G. Annand; Bacteriology, Dr. R. Price; Medicine, Dr. L. Hay.

The meeting adjourned until January 11th, at 9 A.M. The Association met at the Bacteriological Laboratory of the State University, where several autopsies were held for the demonstration of hemorrhagic septicæmia in previously inoculated calves. Dr. Wilson, of the University, who has been making a special study of the disease, gave the Association a talk on the bacteriology of the disease. After dinner the members met at Dr. Price's infirmary for clinics. After the clinical material was disposed of the meeting was again called to order and Dr. Brimhall read his paper on "Hemorrhagic Septicæmia." The paper embraced statistics of the disease, together with full symptoms and post-mortem investigations; the paper was well received by the Association. The following members were appointed to prepare papers for the next meeting: Drs. Ward, Hay, Amos, McDonald, La Pointe, and Price. The meeting then adjourned.

K. J. McKenzie,
Secretary and Treasurer.

